# Efficacy and safety of monoclonal antibodies in the treatment of relapsing remitting multiple sclerosis: a systematic review

**DOI:** 10.1007/s00415-026-13824-y

**Published:** 2026-04-22

**Authors:** Ana Avedillo-Salas, Laura Baeza Martínez, Ana Fanlo-Villacampa, Jorge Vicente-Romero

**Affiliations:** 1 Department of Pharmacology, Physiology and Legal and Forensic Medicine, Faculty of Medicine, University of Zaragoza, 50009 Zaragoza, Spain; 2 Research Network on Global Evidence on Drug Regulation and Safety (GEDRAS), Faculty of Medicine, Institute for Health Research Aragón, University of Zaragoza, 50009 Zaragoza, Spain

**Keywords:** Relapsing–remitting multiple sclerosis, Alemtuzumab, Daclizumab, Ocrelizumab, Ofatumumab, Ublituximab

## Abstract

**Introduction:**

In relapsing–remitting multiple sclerosis (RRMS), conventional immunomodulatory and immunosuppressive therapies are widely used. However, in many cases, optimal control of inflammatory activity and disease progression is not achieved, which has led to the use of biological drugs such as monoclonal antibodies that act specifically on key components of the immune system. The aim was to evaluate the efficacy and safety of monoclonal antibodies compared to other drugs or placebo in adult patients with RRMS.

**Methods:**

A systematic review was performed based on randomized, double-blind, phase III controlled clinical trials published between 2012 and 2025 in the PubMed, Cochrane Library, and Web of Science databases, assessing efficacy and safety in adult patients with RRMS. The review was carried out following the PICO methodology and PRISMA guidelines.

**Results:**

A total of 11 studies were included, evaluating 5 monoclonal antibodies: alemtuzumab, daclizumab, ocrelizumab, ofatumumab, and ublituximab. These therapies showed superior efficacy compared to conventional treatments in reducing the annual relapse rate, MRI inflammatory activity and MRI activity, particularly in patients with highly active disease. However, effects on disability progression were heterogeneous across trials and not consistently significant. In addition, decreases in biomarkers of axonal damage were observed. Nevertheless, relevant adverse effects were identified, including infections, autoimmune reactions, hepatic and cutaneous toxicity, whose incidence varies depending on the drug, requiring close clinical monitoring.

**Conclusions:**

Monoclonal antibodies are an effective option in RRMS, with clinical and radiological benefits superior to those of conventional treatments. Their use requires individualized assessment and close follow-up due to the risk of adverse effects, especially in high-risk patients.

**Supplementary Information:**

The online version contains supplementary material available at 10.1007/s00415-026-13824-y.

## Introduction

Multiple sclerosis (MS) is an autoimmune, chronic inflammatory, neurodegenerative disease of the central nervous system (CNS) that causes demyelination and neurodegeneration. Its origin is multifactorial, involving genetic, environmental, and immunopathogenic factors [1], leading to progressive functional impairments (motor, sensation, vision, and cognition) [2, 3]. The most common symptoms include optic neuritis, brainstem, cerebellar, motor and sensory syndromes, and incomplete transverse myelitis [4, 5], although there are also atypical forms such as bilateral optic neuritis or encephalopathy [6].

The global prevalence of MS is a rising trend that affects approximately 2.8 million individuals worldwide, with a global incidence of 2.1 cases per 100,000 persons/year [7]. Thus, it is the leading cause of non-traumatic disability in young adults (aged 20–40) in the Western world [1].

There are various subtypes of MS including relapsing–remitting MS (RRMS), primary-progressive MS (PPMS), secondary-progressive MS (SPMS), and clinically isolated syndrome (CIS) [8, 9].

RRMS is the most common form of MS, with a higher prevalence in women (3:1) [10]. It is characterized by episodes of neurological deficits (lasting at least 24 h) followed by complete remissions, in which case the symptoms disappear without obvious progression of disability, or partial remissions [11]. If the attacks persist over time, the progression may be less clear [12]. However, in 40% of cases, after 20 years from the initial episode, RRMS can evolve into SPMS due to slow and continuous neurological deterioration that increases the degree of disability regardless of the flare-ups, with a more rapid progression [10–12].

**Fig. 1:**
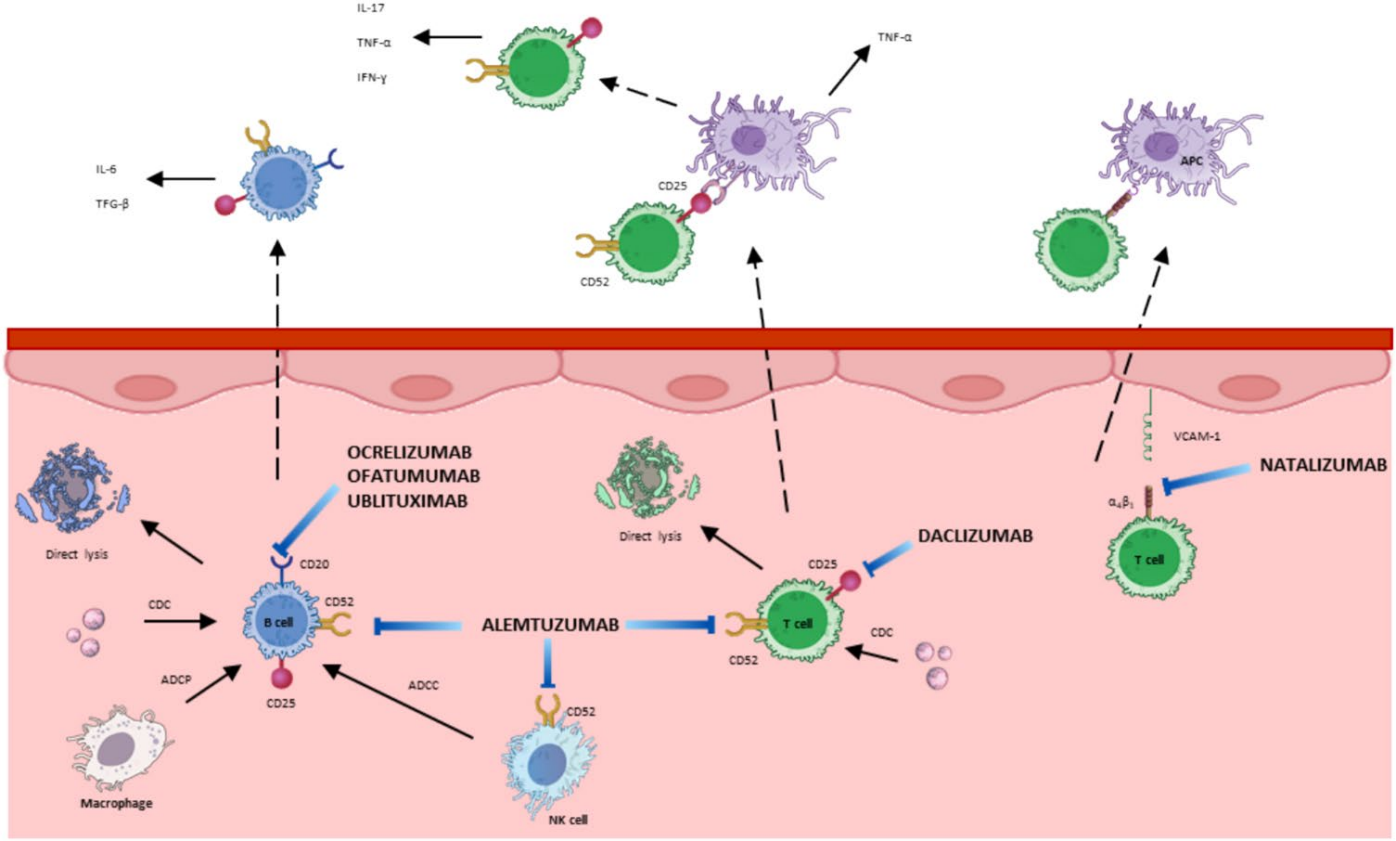
Mechanism of action of monoclonal antibodies in the treatment of RRMS

The current therapeutic approach is based on three fundamental pillars: symptomatic treatment, treatment of flare-ups, and disease-modifying treatments (DMTs). DMTs reduce the frequency of flare-ups, lesion burden and disability progression, forming the basis of current therapy [13]. They include first-line immunomodulatory or immunosuppressive drugs, such as beta interferons, glatiramer acetate, teriflunomide and dimethyl fumarate, which are moderately effective and relatively safe. Second-line drugs, which are more effective but carry a higher risk of adverse effects, include fingolimod, cladribine, and various monoclonal antibodies such as natalizumab, ocrelizumab, ofatumumab, alemtuzumab, ublituximab, and daclizumab, which act on specific immunological targets involved in the pathophysiology of the disease. Most of these drugs are IgG class, and of the IgG1 subclass, while IgA or IgM class drugs are used less frequently due to their reduced accessibility to extravascular regions (Fig. 1). Since the benefits of combination therapy have not been demonstrated, each DMT is used as monotherapy and the choice of drug must be individualized [14, 15].

Since RRMS causes progressive neurodegeneration that leads to a high degree of disability and a reduced quality of life, in the absence of treatment, a high percentage of patients may progress to SPMS, and there is no completely effective treatment. The aim is to evaluate the efficacy and safety of monoclonal antibody drugs compared to placebo or conventional treatment in adult patients diagnosed with RRMS based on clinical trials published between 2014 and 2025.

## Methodology

### Search strategy

The search was conducted using the databases PubMed, the Cochrane Library and Web of Science (WoS). Studies were identified by combining the terms ‘multiple sclerosis’ and ‘relapsing–remitting’ with the keywords antibody, monoclonal, ocrelizumab, ofatumumab, ublituximab, rituximab, natalizumab, alemtuzumab and daclizumab, as well as their drug class. MeSH (Medical Subject Headings) terms and the Boolean operators ‘AND’ and ‘OR’ were also employed. (Supplementary Information Table 1).

**Table 1:** Inclusion criteria based on PICO algorithm

P	Patient	Adult patients (> 18 years) with relapsing–remitting multiple sclerosis
I	Intervention	Treatment with monoclonal antibodies
C	Comparison	Placebo, standard care, any other drug treatment
O	Outcome	Efficacy: Adjusted annualized relapse rate (AARR), No Evidence of Disease Activity (NEDA), Magnetic Resonance Imaging (MRI) (T1 sequence, gadolinium-enhancing lesions, T2 sequence, and brain atrophy), serum levels of light neurofilaments (sNfL), Expanded Disability Status Scale (EDSS), Multiple Sclerosis Functional Composite Scale (MSFC), Symbol Digit Matrix Test (SDMT), Short Form Health Survey (SF-36), Confirmed Disability Accumulation (CDA), Progression independent of relapse activity (PIRA), Relapse-associated worsening (RAW), Confirmed disability worsening (CDW) Safety: incidence and severity of adverse reactions

**Fig. 2:**
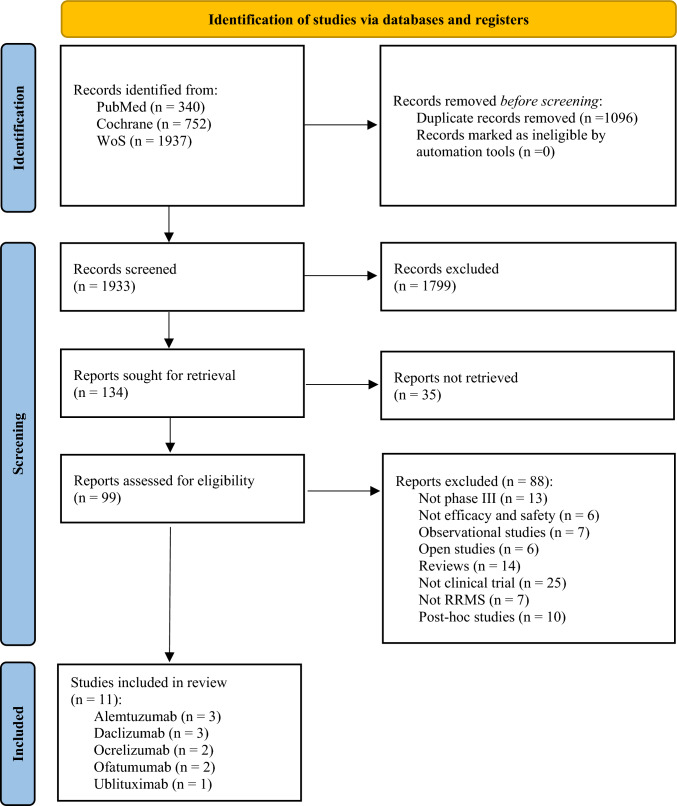
PRISMA flow diagram illustrating the process of selecting studies included in the systematic review. *WoS: Web of Science [16]

This study was conducted according to the PRISMA statement [16]. Articles published between 2012 and 2025 were retrieved. After removing duplicates, titles and abstracts were screened, excluding those that did not meet the inclusion criteria. The remaining records were then assessed for eligibility by careful review of their full texts. A flowchart illustrating the study selection process is shown in Fig. 2.

### Inclusion criteria

Regarding the types of studies, phase III double-blind randomized clinical trials were included. The other inclusion criteria were proposed according to the PICO algorithm (Table 1).

### Exclusion criteria

The proposed exclusion criteria for this systematic review were: (a) studies with insufficient data; (b) in vitro, in silico and in vivo animal studies; (c) commentaries, expert opinions, case reports, letters to the editor, reviews, protocols and study registries, observational studies, systematic reviews and meta-analyses; (d) phase I and II clinical trials; (e) studies that do not include as an intervention at least one of the biologic drugs evaluated in this systematic review; (f) studies with medicinal plants; (g) studies involving patients with Secondary Progressive Multiple Sclerosis (SPMS), Primary Progressive Multiple Sclerosis (PPMS) or mixed populations (i.e., patients with RRMS and progressive MS) and (h) studies that include pregnant or lactating women.

### Data collection and analysis

Data gathering should be conducted by three reviewers independently (A.A.-S., A.F.-V. and J.V.-R). The following information was extracted from each of the included trials: (a) clinical trial registration number, (b) author, (c) publication date, (d) trial design, (e) participant characteristics, (f) interventions delivered with each dose regimen, (g) comparison group dose regimen, (h) age of participants, and (i) outcomes. The extracted data were entered into an excel spreadsheet and cross-checked by three authors (L.B.-M., A.F.-V. and J.V.-R), and the senior author (A.A.-S) was involved in the discussion and decision-making in case of disagreement during data extraction.

### Quality assessment and risk of bias

The quality of evidence was assessed following the Grading of Recommendations Assessment, Development and Evaluation (GRADE) system [17] (Supplementary Information Table 2).

**Table 2:** Alemtuzumab efficacy results

References	No. of participants	Results Alemtuzumab vs. IFN-β1a
Cohen et al., 2012 [19]	IG: n = 376 CG: n = 187	Relapse-free patients (%): 77.6 vs. 58.7; *p* < 0.0001 Sustained accumulation confirmed 6 m (%): 8 vs. 11 Mean change in EDSS score from baseline: EDSS: -0.14 vs. -0.14; *p* = 0.97 Change in MSFC score from baseline: + 0.15 vs. + 0.07; *p* = 0.01 Patients with gadolinium-enhancing lesions at 24 m (%): 7 vs. 19; *p* < 0.0001 Patients clinically disease-free (%): 74 vs. 56; *p* < 0.0001 Patients MRI and clinically disease-free (%): 39 vs. 27; *p* = 0.006
Kuhle et al., 2021 [20]	IG: n = 376 CG: n = 187	*Median sNfL levels (pg/mL)*: Baseline: 31.7 vs. 31.4 *p* = 0.57 6 m: 17.2 vs. 21.4 *p* < 0.0001 24 m: 13.2 vs. 18.7 *p* < 0.0001
Giovannoni et al., 2016 [21]	IG: n = 426 CG: n = 202	Worsening EDSS baseline-24 m (% of patients): 8 vs. 13 Worsening ≥ 15% according to MSFC 24 m (%): 18.7 vs. 27.6 Confirmed improvement in disability 6 m (Kaplan–Meier) (%): 28.8 vs. 12.9; *p* = 0.0002 *Clinical improvement for 7 functional systems assessed by EDSS 24 m*: Cerebral: OR (95% CI) = 2.54 (1.76–3.69); *p* < 0.0001 Cerebellar: OR (95% CI) = 2.26 (1.60–3.20); *p* < 0.0001 Sensory: OR (95% CI) = 1.88 (1.34–2.65); *p* = 0.0003 Pyramidal: OR (95% CI) = 1.62 (1.16–2.25); *p* = 0.0044 Visual: OR (95% CI) = 1.59 (1.11–2.30); *p* = 0.0122 Brainstem: OR (95% CI) = 1.43 (0.99–2.07); *p* = 0.0597 Bowel/bladder: OR (95% CI) = 1.37 (0.95–1.96); *p* = 0.0914

*CI* confidence interval, *CG* control group, *EDSS* expanded disability status scale, *IG* intervention group, *m* months, *MRI* magnetic resonance imaging, *MSFC* multiple sclerosis functional composite, *OR* odds ratio, *sNfL* serum levels of light neurofilaments

Risk of bias was assessed using the RoB2 [18]. Each study was independently rated by three researchers (A.A.-S., A.F.-V. and J.V.-R), with disagreements resolved using the more conservative score. Overall, the risk of bias was considered low across all domains, particularly in domains related to randomization and outcome assessment, reflecting the high methodological quality of phase III randomized controlled trials included in this review (Supplementary Information Fig. 1).

## Results

### Alemtuzumab

The drug alemtuzumab was evaluated in three studies arising from the CARE-MS I and CARE-MS II clinical trials, both of which were blinded for evaluators and lasted two years.

The CARE-MS I trial was analyzed by Cohen et al. [19] and Kuhle et al. [20] and included patients aged 18 to 50 years with RRMS without prior disease-modifying treatment, with a baseline EDSS score between 0 and 3, randomized 2:1 to IV alemtuzumab (12 mg/day, 5 days at the start and 3 days per year) or SC interferon β1a (44 μg three times/week) for 104 weeks. CARE-MS II included patients aged 18 to 55 years with RRMS previously treated with DMT and who had relapses during that treatment, with EDSS values between 0 and 3, and randomized to the same treatment regimen.

In the study by Cohen et al. [19], 77.6% of patients treated with alemtuzumab remained relapse-free (*p* < 0.0001) and had less disability progression (8%) compared with IFN-β1a. Although the mean reduction in the EDSS scale was similar between groups, the MSFC score improved significantly with alemtuzumab (*p* = 0.01). Radiologically, this group showed a greater reduction in T2 lesion volume, a lower proportion of new T2 lesions (45% vs. 58%; *p* = 0.04) and fewer gadolinium-enhancing lesions (7% vs. 19%; *p* < 0.0001), as well as significantly less brain loss. Disease-free survival was higher with alemtuzumab (74% vs. 56%; *p* < 0.0001), and 39% remained free of clinical and radiological activity compared to 27% in the control group (Table 2).

In terms of safety, adverse events (AEs) were common in both groups (96% alemtuzumab vs. 92% IFN-β1a), with serious effects in 18% and 14%, respectively. However, treatment discontinuation was lower with alemtuzumab (1% vs. 6%). In the trial, more infections (67% vs. 45%) and infusion reactions (90%) were observed with alemtuzumab, as well as thyroid disorders (18% vs. 6%), including two cases of carcinoma. Haematological disorders were similar (18% vs. 19%), while liver toxicity was lower (4% vs. 17%). A detailed overview of the adverse events by individual monoclonal antibody is presented in Supplementary Information (Table 3).

**Table 3:** Efficacy results of Daclizumab vs Interferon-β1a

References	No. of participants	Efficacy (Daclizumab vs. IFN-β1a)
Kappos et al., 2015 [22]	IG: n = 919 CG: n = 922	*Adjusted annualized relapse rate*: 0.22 (0.19–0.24) vs. 0.39 (0.35–0.44); *p* < 0.001 *Proportion of patients free from relapse at 144w:* Estimated percentage ∫ (%): 67 vs. 51; *p* NR HR: 0.59 (0.50–0-69) *p* NR *New or increased T2 lesions at 96w:* Adjusted mean no. (95%CI): 4.3 (3.9–4.8) vs. 9.4 (8.5–10.5); *p* < 0.001 Percent Reduction (95% CI): 54 (47–61); *p* NR *Clinically significant worsening (MSIS-29) 96 s:* Estimated percentage (%): 19 vs. 23; *p* NR Percent reduction in the Odds (95%CI): 24 (5–40); *p* NR
Benedict et al., 2018 [23]	IG: n = 919 CG: n = 922	*Initial SDMT score:* Mean (SD): 48.5 (15.9) vs. 47.7 (16.1) Median (range): 49 (0–110) vs. 49 (3–110) *Improvement SDMT* w96: Mean (SD): 4.1 (12.4) vs. 2.9 (12.7); *p* = 0.0274 ≥ 3 points (%): 60 vs. 54.1; *p* = 0.0153 ≥ 4 points (%): 55.4 vs. 50.1; *p* = 0.0366 *Improvement SDMT w*144: Mean (SD): 6.3 (12.6) vs. 3.1 (13.2); *p* = 0.0024 ≥ 3 points (%): 65.5 vs. 52; *p* = 0.0028 ≥ 4 points (%): 61.7 vs. 48.4; *p* = 0.0067

*CI* confidence interval, *CG* control group, *EDSS* expanded disability status scale, *HR* hazard ratio, *IG* intervention group, *MSIS-29* multiple sclerosis impact scale, *p NR* p value not reported, *SDMT* symbol digit matrix test, *w* week, *∫* the percentage of patients was estimated by means of Kaplan–Meier product-limit method

Kuhle et al. [20] evaluated serum levels of light neurofilaments as biomarkers of axonal damage. Baseline levels were comparable between groups, but at six months, a significant decrease was observed with alemtuzumab versus interferon (17.2 vs. 21.4 pg/mL; *p* < 0.0001), which was maintained at 18 months (13.2 vs. 15.6 pg/mL; *p* = 0.01). At 24 months, levels stabilised with alemtuzumab, while they increased with interferon. In the subgroup with highly active disease, sNfL levels were initially higher, although the decrease was equally greater with alemtuzumab (*p* < 0.05 at all follow-up points) (Table 2).

For their part, Giovannoni et al. [21] analyzed the CARE-MS II trial, comparing the efficacy of alemtuzumab and interferon using the EDSS and MSFC scales at 24 months. Alemtuzumab showed greater improvement in disability, with a higher percentage of patients experiencing reductions of one point or more in the EDSS score (14% vs. 10%). Significant clinical worsening was less frequent in the alemtuzumab group (5% vs. 9%), while the proportion of patients with stable EDSS scores was similar between the two treatments. The EDSS functional domain analysis showed a higher probability of significant improvement with alemtuzumab in the mental, cerebellar, sensory, pyramidal, and visual systems. Although improvements in the brainstem and bladder/bowel did not reach statistical significance, the trend was equally favourable for alemtuzumab According to the MSFC scale, worsening ≥ 15% at 24 months was lower with alemtuzumab than with interferon. Finally, Kaplan–Meier analysis estimated a probability of sustained improvement in disability at six months of 28.8% versus 12.9%, thus confirming the superiority of the monoclonal antibody in both clinical and radiological efficacy (Table 2).

### Daclizumab

Daclizumab was evaluated in three studies of the DECIDE trial, which was multicenter, quadruple-masked, and lasted 144 weeks. Patients aged 18–55 years with an EDSS score of 0.0–5.0 were included. Participants were randomized (1:1) to receive daclizumab or IFN-β1a. The experimental group received daclizumab HYP SC 150 mg every 4 weeks and weekly IM placebo, while the control group received IFN-β1a SC 30 μg weekly and SC placebo every 4 weeks.

Kappos et al. [22] reported that the annualized rate of flare-ups was significantly lower with daclizumab than with IFN-β1a (0.22 vs. 0.39; *p* < 0.001). After 144 weeks, 67% of treated patients remained relapse-free and there was a 41% reduction in the risk of relapse. Radiologically, daclizumab significantly reduced the number of new or enlarged T2 lesions (4.3 vs. 9.4; *p* < 0.001), corresponding to a 54% reduction in lesion progression. In terms of functional deterioration (MSIS-29), 19% of patients on daclizumab showed clinically significant worsening compared to 23% on IFN-β1a, with a relative reduction of 24% in the probability of deterioration (Table 3).

In terms of safety, adverse events were frequent and similar between groups (91%). Serious events were slightly more frequent with daclizumab (24% vs. 21%, excluding relapses 15% vs. 10%), as were infections (65% vs. 57%) and skin effects (37% vs. 19%). Treatment discontinuation due to adverse events occurred in 15% with daclizumab and 12% with IFN-β1a. Hepatic events were comparable, and cases of neoplasms without a direct causal relationship were reported.

The study by Benedict et al. [23] assessed cognitive efficacy using SDMT. The mean baseline score was comparable between groups (48.5 vs. 47.7). At 96 weeks, improvement was significantly greater with daclizumab (+ 4.1 vs. + 2.9 points; *p* = 0.02), with a higher percentage of patients achieving increases equal to or greater than 3 and 4 points. This trend was maintained at 144 weeks (Table 3).

As for the study by Krueger et al. [24], it reported that 37% of patients treated with daclizumab and 19% treated with interferon experienced some type of dermatological event, predominantly dermatitis (14% vs. 6%), eczema (10% vs. 4%), and rash (3% vs. 1%). Although most cases were mild or moderate, 50 patients (43 with daclizumab and 7 with IFN-β1a) discontinued treatment and 19 patients (15 and 4, respectively) withdrew from the study.

### Ocrelizumab

Hauser et al. [25] evaluated the drug ocrelizumab through the OPERA I and II clinical trials, which employed double simulation and triple masking. Patients were randomly assigned (1:1) to receive either IV ocrelizumab or subcutaneous (SC) IFN-β1a. The experimental group received an initial dose of 300 mg of IV ocrelizumab on days 1 and 15, followed by 600 mg infusions every 24 weeks, in addition to a SC placebo three times a week. The control group received 44 μg of SC IFN-β1a three times per week and an IV placebo on days 1 and 15, followed by an infusion every 24 weeks.

Ocrelizumab significantly reduced clinical and radiological activity compared to interferon. The annualized relapse rate was 0.16 versus 0.29 in both trials (RR = 0.53–0.54; *p* < 0.001), representing a 46% reduction in relapse risk. Confirmed disability progression at 12 weeks was observed in 7.6% and 10.6% of patients treated with ocrelizumab in OPERA I and OPERA II, respectively, compared with 12.2% and 15.1% of patients treated with IFN-β1a. This represented a 43% and 37% reduction in risk, respectively, and the trend continued at 24 weeks. Confirmed disability improvement was also more frequent with ocrelizumab. No evidence of disease activity (NEDA) at 96 weeks was achieved by 47.9% and 47.5% of patients treated with ocrelizumab in OPERA I and OPERA II respectively, compared to 29.2% and 25.1% of patients treated with IFN-β1a (*p* < 0.001) (Table 4).

**Table 4:** Clinical efficacy results of ocrelizumab versus IFN-β-1a Hauser et al. [25]

	OPERA I Trial			OPERA II Trial				
	Ocrelizumab (N = 410)	IFN-β1a (N = 411)	*p* value	Ocrelizumab (N = 417)			IFN-β1a (N = 418)	*p* value
Primary end point								
Annualized relapse rate (96 wk)	0.16	0.29		0.16			0.29	
Rate ratio (95% CI)	0.54 (0.40–0.72)		*p* < 0.001	0.53 (0.40–0.71)				*p* < 0.001
Disability progression confirmed (12 wk)								
Patients with event (%)	7.6	12.2		10.6			15.1	
HR (95% CI)	0.57 (0.37–0.90)		*p* = 0.01	0.63 (0.42–0.92)				*p* = 0.02
Disability progression confirmed (24 wk)								
Patients with event (%)	5.9	9.5		7.9			11.5	
HR (95% CI)	0.57 (0.34–0.95)		*p* = 0.03	0.63 (0.40–0.98)				*p* = 0.04
Disability improvement confirmed (12 wk)								
Patients with event (%)	20.0	12.4		21.4			18.8	
Difference (%)	61*		*p* = 0.01	14*				*p* = 0.40
No evidence of disease activity (96 wk)								
Patients (%)	47.9	29.2		47.5			25.1	
Difference-% (95% CI)	64 (36–98)		*p* < 0.001	89 (54–132)				*p* < 0.001
MSFC score (96 wk)								
Adjusted mean score	0.21	0.17		0.28			0.17	
Difference-% (95% CI)	0.04 (− 0.04–0.12)		*p* = 0.33	0.11(0.03–0.18)				*p* = 0.004
Change in SF-36 physical-component (96 wk)								
Adjusted mean score	+ 0.04	− 0.66		+ 0.33			− 0.83	
Difference-% (95% CI)	0.69 (− 0.41–1.80)		*p* = 0.22	1.16 (0.05–2.27)				*p* = 0.04

*CI* confidence interval, *HR* hazard ratio, *IFN-β1a* interferon-β1a, *MSFC* multiple sclerosis functional composite, *SF-36* short form-36 health survey, * 95% CI not reported

With regard to radiological criteria, ocrelizumab demonstrated a substantial reduction in inflammatory activity. In OPERA I, gadolinium-enhancing lesions on T1 were detected in 8.3% of patients treated with ocrelizumab compared to 30.2% with interferon (RR = 0.06; *p* < 0.001), while new or enlarged lesions on T2 affected 38.3% and 61.3%, respectively (RR = 0.23; *p* < 0.001). Furthermore, the study revealed that brain volume loss from week 24 to week 96 was lower with ocrelizumab than with interferon, with a respective loss of -0.57% and -0.74%. In the OPERA II, gadolinium-enhancing lesions were observed in 9.8% of patients treated with ocrelizumab compared to 31.6% of those treated with interferon (*p* < 0.001). New T2 lesions were also noted in 39.1% of the ocrelizumab group compared to 62.0% of the interferon group (RR = 0.17; *p* < 0.001). The study found that there was a significant decrease in brain volume, with ocrelizumab resulting in a loss of -0.64% and interferon causing a loss of -0.75% (*p* = 0.009).

In terms of safety, the overall incidence of adverse events was comparable between groups in both trials. In the OPERA I, AEs were reported in 80.1% of patients receiving ocrelizumab and 80.9% receiving interferon, while in OPERA II, the values were 86.3% and 85.6%, respectively. However, the discontinuation rate due to AEs was lower with ocrelizumab (3.2% and 3.8%) than with interferon (6.4% and 6%). Serious AEs were observed with a lower frequency in ocrelizumab (6.9–7%) than in interferon (7.8–9.6%) treatment groups. Infusion-related reactions were observed to occur with significantly greater frequency in patients receiving ocrelizumab (30.9% in OPERA I and 37.6% in OPERA II) in comparison to those administered interferon (7.3% and 12%), although it should be noted that the majority of these reactions were classified as mild or moderate. Furthermore, infections were reported in more than half of patients (56.9–60.2% ocrelizumab vs. 52.5–52.8% interferon), with herpes zoster (1.9–2.2%) and oral herpes (2.2–3.6%) being slightly more prevalent in the ocrelizumab group than in the interferon group (1% and 2–2.2%, respectively). However, serious infections were observed with a lower frequency in the monoclonal antibody group (1.2–1.4%) than in the interferon group (2.9%). Neoplasms manifested infrequently and similarly across the two groups (0.7% vs. 0.2% in OPERA I).

Kappos et al. [26], which drew on both trials, examined the contribution of relapse-independent progression (PIRA) and relapse-associated worsening (RAW) to confirmed cumulative disability. At the 12-week mark, the CDA was recorded at 21.1% in the ocrelizumab group as compared to 29.6% in the interferon group. Similarly, at the 24-week point, the CDA stood at 16.2% in the ocrelizumab group as against 22.7% in the interferon group, thereby substantiating a substantial decline in risk. PIRA was responsible for the majority of CDA events, with 88% of CDA events in the ocrelizumab group at 12 weeks and 89.1% at 24 weeks being attributable to PIRA, as opposed to 78% with interferon. The occurrence of RAW events was found to be less frequent in the experimental group than in the control group (3% vs. 6.2% at 12 weeks; 2.1% vs. 4.8% at 24 weeks).

### Ofatumumab

Ofatumumab was evaluated by Hauser et al. [27] and Gärtner et al. [28] in the ASCLEPIOS I and II clinical trials. Both trials were randomized, double-blind, double-dummy, active-controlled, multicenter trials conducted in adult patients aged 18 to 55 years with an EDSS between 0.0 and 5.5.

In the analysis by Hauser et al. [27], ofatumumab demonstrated significant superiority over teriflunomide in terms of clinical and radiological efficacy. As demonstrated in the ASCLEPIOS study, the annualized relapse rate was 0.11 in the ofatumumab-treated group, in comparison with 0.22 in the teriflunomide-treated group (*p* < 0.001). Similarly, in ASCLEPIOS II, annualized rates of 0.10 and 0.25 were observed, respectively (*p* < 0.001). The risk of confirmed disability progression at three months was reduced by 35% in ASCLEPIOS I and by 34% in ASCLEPIOS II in the ofatumumab group. At the six-month mark, the risk reduction was 39% in ASCLEPIOS I (HR = 0.61; *p* = 0.01) and 24% in ASCLEPIOS II (HR = 0.76; *p* = 0.13). The proportion of patients demonstrating sustained improvement in disability was higher in the ofatumumab-treated group than in the control group in both studies (ASCLEPIOS I: 9.7% vs. 8.2%; ASCLEPIOS II: 12.3% vs. 8.1%), although these differences did not reach statistical significance. With regard to the radiological criteria, ofatumumab demonstrated a significant decrease in inflammatory activity. The mean number of gadolinium-enhancing T1 lesions was 0.01 vs. 0.45 in ASCLEPIOS I and 0.03 vs. 0.51 in ASCLEPIOS II, with relative reductions of 97% and 94% (*p* < 0.001). Conversely, new or enlarged T2 lesions exhibited a marked decrease (RR = 0.18 and 0.15; *p* < 0.001), and annual brain volume loss demonstrated comparable trends between the groups. Serum NfL levels were lower with ofatumumab, reflecting less axonal damage (8.8–6.9 pg/mL vs. 9.4–9.0 pg/mL in ASCLEPIOS I; *p* ≤ 0.01 in all cases) (Table 5).

**Table 5:** Efficacy results of ofatumumab and ublituximab versus teriflunomide

	ASCLEPIOS I [27]			ASCLEPIOS II [27]			RDTN [28]			ULTIMATE I [29]			ULTIMATE II [29]		
	Ofatumumab (N = 465)	Teriflunomide (N = 462)	*p* value	Ofatumumab (N = 481)	Teriflunomide (N = 474)	*p* value	Ofatumumab (N = 314)	Teriflunomide (N = 301)	*p* value	Ublituximab (N = 271)	Teriflunomide (N = 274)	*p* value	Ublituximab (N = 272)	Teriflunomide (N = 272)	*p* value
Primary end point															
AARR	0.11	0.22	*p* < 0.001	0.1	0.25	*p* < 0.001	0.09	0.18	*p* < 0.001	0.08	0.19	NR	0.09	0.18	NR
RR (95% CI)	0.49 (0.37–0.65)		*p* < 0.001	0.42 (0.31–0.56)		*p* < 0.001	0.5 (0.33–0.74)		*p* < 0.001	0.41(0.27–0.62)		*p* < 0.001	0.51 (0.33–0.78)		*p* = 0.002
3mCDW															
% of events during the trial/no. of participants	11.3	15.4		10.5	14.6		7.7	12.3							
HR (95% CI)	0.65 (0.45–0.96)		NR	0.66 (0.45–0.97)		NR	0.62 (0.37–1.03)		*p* = 0.065						
6mCDW															
% of events during the trial/no. of participants	8.2	13.0		8.0	10.9		5.4	10.0							
HR (95% CI)	0.61 (0.40–0.93)		NR	0.76 (0.49–1.17)		NR	0.54 (0.30–0.98)		*p* = 0.044						
Worsening on SDMT from baseline to 96wk															
% of participants										29.2	31.8		29.0	31.6	
OR (95% CI)										0.87 (0.60–1.26)		NR	0.86 (0.60–1.25)		NR
Disability improvement confirmed (6 m)															
No. of events during the trial/no. of participants	33/375	26/363		41/374	27/360										
HR (95% CI)	1.19 (0.71–1.98)		NR	1.52 (0.93–2.47)		NR									
Gadolinium-enhancing lesions per T1-weighted MRI scan															
Mean	0.01	0.45		0.03	0.51		0.02	0.39		0.02	0.49		0.01	0.25	
RR (95% CI)	0.03 (0.01–0.05)		*p* < 0.001	0.06 (0.04–0.10)		*p* < 0.001	0.05 (0.02–0.10)		*p* < 0.001	0.03 (0.02–0.06)		*p* < 0.001	0.04 (0.02–0.06)		*p* < 0.001
New or enlarging hyperintense lesions per T2-weighted MRI scan															
Mean	0.72	4.00		0.64	4.15		0.86	4.78		0.21	2.79		0.28	2.83	
RR (95% CI)	0.18 (0.15–0.22)		*p* < 0.001	0.15 (0.13–0.19)		*p* < 0.001	0.18 (0.14–0.24)		*p* < 0.001	0.08 (0.06–0.10)		*p* < 0.001	0.10 (0.07–0.14)		*p* < 0.001
Change in brain volume from baseline															
Least-squares mean	− 0.28	− 0.35	*p* = 0.12	− 0.29	− 0.35	*p* = 0.13	− 0.30	− 0.30	*p* = 0.9	− 0.19	− 0.18	NR	− 0.19	− 0.18	NR
sNfL concentration geometric mean (pg/mL)															
3 m 12 m 24 m	8.8 7.0 6.9	9.4 9.6 9.0	*p* = 0.01 *p* < 0.001 *p* < 0.001	8.9 7.1 6.8	10 9.5 9.0	*p* < 0.001 *p* < 0.001 *p* < 0.001	8.72 6.60 6.47	9.13 8.61 8.10	*p* < 0.258 *p* < 0.001 *p* < 0.001						
NEDA-3															
% of participants							0–12 m: 47 12–24 m: 92.1 0–24 m: 44.6	24.7 46.8 17.7		24–96 wk: 44.6	15		24–96 wk: 43.0	24–96 wk: 11.4	
OR (95% CI)							0–12 m: 3.31 (2.24–4.90) 12–24 m: 14.68 (8.76–24.61) 0–24 m: 4.63 (3.05–7.03)		*p* < 0.001 *p* < 0.001 *p* < 0.001	24–96 wk: 5.44 (3.54–8.38)		NR	24–96 wk: 7.95 (4.92–12.84)		NR

*AARR* adjusted annualized relapse rate**,** *CI* confidence interval, *CDW* confirmed disability worsening, *HR* hazard ratio, *MRI* magnetic resonance imaging, *m* months, *NEDA* no evidence of disease activity, *NR* Not reported, *OR* odds ratio, *RDTN* recently diagnosed treatment-naïve, *RR* rate ratio, *SDMT* symbol digit modalities test, *sNfL* serum levels of light neurofilaments, wk week

With regard to safety, the overall incidence of adverse events was comparable between the two groups. In ASCLEPIOS I, 82.2% of those treated with ofatumumab and 82.3% with teriflunomide experienced an event, with discontinuations of 5.8% and 5.6%, respectively. Infections were observed to be marginally less prevalent in the ofatumumab group (49.2% vs. 51.5%), although serious infections were found to be somewhat more prevalent in the latter group (2.6% vs. 1.5%). Systemic reactions to subcutaneous administration were comparable (16.1% vs. 16.5%), with two serious cases (0.4%) documented in the experimental group. In ASCLEPIOS II, the frequency of adverse events was 85% vs. 86.1%, with treatment discontinuation in 5.6% and 5.3%, respectively. Serious infections occurred in 2.5% and 2.1% of patients, respectively, and systemic reactions were more common with ofatumumab (24.1% vs. 13.5%), although none were considered serious. Neoplasms were rare (≤ 0.6%) and were not considered treatment-related.

Gärtner et al. [28] demonstrated that in a subgroup of newly diagnosed patients without prior treatment (RDTN), there was a lower annualized relapse rate with ofatumumab than with teriflunomide (RR = 0.50; *p* < 0.001). The findings demonstrated that confirmed disability progression was lower with ofatumumab at three months and reached a level of statistical significance at six months (HR = 0.54; *p* = 0.004). The study revealed that gadolinium-enhancing T1 lesions were significantly lower (0.02 vs. 0.39; RR = 0.05; *p* < 0.0001), as were new or growing T2 lesions (0.86 vs. 4.78; RR = 0.18; *p* < 0.001). Conversely, the proportion of patients exhibiting no disease activity was notably higher in the ofatumumab group across all three assessment points (6, 12, and 24 months). Moreover, serum NfL levels exhibited a progressive decline in the experimental group in comparison to the control group (*p* < 0.001) (Table 5).

With regard to safety, the frequency of adverse events was comparable (84.7% vs. 86%), although discontinuation due to adverse events was marginally higher with ofatumumab (6.1% vs. 2.3%). The most frequently reported effects were nasopharyngitis, local reactions, headache, and respiratory infections, which were generally mild. Serious events (7% vs. 5.3%) included infections (1.9% vs. 0.7%) and neoplasms (0.6% vs. 0.3%), with no deaths reported.

### Ublituximab

Ublituximab has been the subject of analysis in a study by Steinman et al. [29], which is based on the ULTIMATE I and ULTIMATE II clinical trials. The two trials were conducted in adult patients aged between 18 and 55 years with an EDSS between 0.0 and 5.5.

With regard to the question of efficacy, the ULTIMATE I trial demonstrated a lower annualized relapse rate with ublituximab than with teriflunomide, exhibiting a relative reduction of 56% (RR = 0.44; *p* < 0.001). In ULTIMATE II, the values were 0.09 vs. 0.18, with a 49% reduction in the risk of relapse with ublituximab. With regard to the radiological results, at week 96, the mean number of gadolinium-enhancing lesions was found to be significantly lower with ublituximab (0.02 vs. 0.49 in ULTIMATE I; 0.01 vs. 0.25 in ULTIMATE II; *p* < 0.001). In a similar vein, new or enhanced lesions on T2-weighted sequences exhibited a 90–92% reduction in the risk of new inflammatory lesions. The loss of brain volume was found to be similar between the two groups, with a decrease of − 0.19% in the ublituximab group and − 0.18% in the teriflunomide group. The absence of evidence of disease activity between weeks 24 and 96 was significantly more prevalent in the ublituximab group (44.6%) than in the teriflunomide group (15%) in both trials (*p* < 0.001) (Table 5).

With regard to safety, the proportion of patients experiencing adverse events was high and similar between groups (86–93%), although a higher frequency of infusion-related reactions was observed with ublituximab (44–51% vs. 7–18%) and a slight increase in the incidence of serious infections (4–5% vs. 2–3%). Neoplasms were uncommon, with a prevalence of less than 1%, and three deaths were reported, possibly related to treatment.

## Discussion

The results analyzed in this review showed that monoclonal antibodies represent a significant advance in the treatment of RRMS, exhibiting superior efficacy compared to conventional disease-modifying treatments, both in controlling inflammatory activity and in reducing cumulative neurological damage. This superiority has been observed in multiple dimensions of the disease, including a reduction in relapses, radiological activity as assessed by magnetic resonance imaging, disability progression and, in some cases, biomarkers of neurodegeneration.

In this context, alemtuzumab is distinguished by its markedly superior clinical and radiological efficacy in comparison with IFN-β1a, both in naïve patients and in those with previous therapeutic failure. The substantial decrease in relapses and subclinical inflammatory activity evident in the studies conducted by Cohen et al. [19] indicates that the disease is being effectively and consistently managed, a finding of particular relevance in the initial phases of RRMS. The reduced loss of brain volume, coupled with the increased proportion of patients demonstrating no clinical or radiological activity, lends further support to the hypothesis that rigorous inflammatory control during the early stages of the condition may lead to a reduction in long-term structural damage.

The higher proportion of patients demonstrating no clinical or radiological activity lends further support to the hypothesis that the early use of highly effective therapies has the capacity to modify the natural course of the disease, thereby reducing the accumulation of irreversible damage from the early stages. In this regard, alemtuzumab is positioned as a particularly attractive option in patients with recent diagnosis and high inflammatory activity, in whom early control could translate into better long-term functional outcomes.

The analysis of serum neurofilament provides further evidence that contributes to the interpretation of these results. The substantial and enduring decrease in sNfL with alemtuzumab, particularly in patients with highly active disease, indicates a direct effect on axonal damage, extending beyond the mere control of relapses. This finding supports the hypothesis of a possible neuroprotective effect, in accordance with the reduced brain atrophy observed, and reinforces its indication in patients at high risk of early progression.

With regard to disability, the results highlight the limitations inherent in the EDSS scale, which may not be sensitive enough to detect functional changes in the short to medium term. Conversely, the MSFC scale exhibited substantial enhancements in the alemtuzumab-treated cohort, particularly in the motor and cognitive domains, suggesting a more comprehensive functional recovery. This discrepancy underscores the necessity of employing multidimensional scaling methodologies to accurately assess the true impact of highly effective treatments. In patients with previous treatment failure, the higher probability of confirmed disability improvement observed in the studies by Giovannoni et al. [21] reinforces the value of alemtuzumab as a rescue option in active RRMS.

However, this high efficacy is accompanied by a more complex safety profile than that of conventional DMTs. The higher frequency of infections, infusion reactions, thyroid disorders, and liver toxicity observed in this study reflects their potent immunosuppressive effect. It is acknowledged that such occurrences are, to a certain extent, anticipated on the basis of their underlying mechanisms. However, their manifestation gives rise to the initiation of rigorous and protracted monitoring programmes encompassing vital signs and hepatic function. Consequently, pharmacovigilance alerts issued by the AEMPS and EMA, which include serious cardiovascular and immune-mediated events [30, 31], emphasize the necessity for meticulous patient selection and comprehensive clinical monitoring during and after drug administration. Although rare, the occurrence of thyroid neoplasms underscores the necessity for long-term follow-up in highly effective therapies. Nevertheless, the low discontinuation rate observed indicates that, under appropriate monitoring conditions, alemtuzumab may be well tolerated overall.

In relation to daclizumab, the DECIDE trial [22, 23] demonstrated clear superiority over IFN-β1a in reducing flare-ups, radiological progression and cognitive impairment, which initially positioned this drug as a promising option. Nevertheless, the high incidence of severe adverse effects [22, 24], particularly cutaneous, hepatic, and neurological manifestations, constrained its clinical applicability. The withdrawal of daclizumab from the market following reports of cases of inflammatory encephalitis and meningoencephalitis highlights the critical importance of post-marketing pharmacovigilance [32, 33]. This underscores the necessity for the complementation of efficacy evidence derived from controlled clinical trials with long-term safety data derived from real-world clinical practice, particularly in the context of intensive immunomodulatory treatments.

Monoclonal antibodies directed against CD20 (ocrelizumab, ofatumumab and ublituximab) demonstrate a high and relatively homogeneous efficacy profile, characterized by almost complete suppression of acute inflammation as evidenced by MRI.

Ocrelizumab has been shown to result in a reduction in flare-ups, a slower rate of disability progression, and superior radiological control in comparison with IFN-β1a [25, 26], without causing a significant increase in overall risk. A detailed analysis of the various components of disability progression has been conducted, and the results of this analysis suggest that this drug is effective in reducing flare-associated activity. It is therefore concluded that the main residual mechanism is now relapse-independent progression. This finding could indicate a more profound impact on central inflammatory mechanisms and a potential disease-modifying effect.

Ofatumumab has been demonstrated to demonstrate superior clinical efficacy in comparison with teriflunomide, in both previously treated and newly diagnosed patients [27, 28]. The observed reduction in the incidence of flare-ups, the high proportion of patients exhibiting no evidence of disease activity (NEDA), in conjunction with the sustained reduction in soluble neurofilament light, suggests a prolonged inflammatory response and a potential neuroprotective effect. However, discrepancies observed between studies in terms of tolerability and discontinuation indicate that its safety profile should be interpreted with caution and in the context of the clinical setting, as it may be influenced by contextual factors and the baseline characteristics of patients, reinforcing the need for individualized monitoring.

In addition, ublituximab [29] has been demonstrated to demonstrate high efficacy in the control of acute inflammation, flare-ups and radiological activity, with superiority over teriflunomide, achieving almost complete suppression of gadolinium-enhancing lesions. However, the absence of clear benefits in disability and cognitive function could indicate a more limited effect on underlying neurodegenerative processes, at least in the short term. Moreover, the elevated rate of discontinuation and the occurrence of serious infections indicate the necessity for further evaluation of its long-term safety profile, despite its observed efficacy.

The observed differences in efficacy and safety profiles among monoclonal antibodies may be partly explained by their distinct mechanisms of action. Anti-CD20 therapies (ocrelizumab, ofatumumab, and ublituximab) selectively deplete B lymphocytes, leading to a sustained reduction in inflammatory activity, particularly reflected in MRI outcomes. In contrast, alemtuzumab induces a broader immune reconstitution by targeting CD52, resulting in long-term immune modulation but also a higher risk of secondary autoimmunity. Daclizumab, which targets the IL-2 receptor (CD25), modulates T-cell activation but has been associated with significant immune-mediated adverse events, ultimately leading to its withdrawal from clinical use. These mechanistic differences may explain variations in both efficacy magnitude and safety risks and should be considered when individualizing treatment strategies.

Accordingly, treatment selection should be guided not only by efficacy and safety outcomes but also by patient-specific risk factors, including age, comorbidities, prior treatments, and individual infection risk. Thus, comprehensive risk management strategies should include baseline screening (e.g., infections and autoimmune markers) and regular monitoring for infections, malignancies, and autoimmune complications to optimize the benefit-risk balance.

It is imperative to acknowledge the limitations of this systematic review, particularly regarding the direct clinical applicability of our findings. The dearth of available clinical trials, the heterogeneity of the studied populations, and the absence of direct head-to-head comparisons between monoclonal antibodies all serve to make it difficult to draw definitive conclusions about which therapy possesses the optimal benefit-risk profile. Moreover, although the individual evaluation of the included pivotal trials yielded a low risk of bias in the randomization process and deviations from intended interventions, the overall assessment of the evidence using the GRADE framework necessitated downgrading the final certainty to a high/moderate level. Discrepancies in administration routes and study designs have the potential to engender additional biases in the comprehensive evaluation of both efficacy and safety. Consequently, this variability must be carefully considered when interpreting the findings and their generalizability.

## Conclusions

Monoclonal antibodies analyzed have showed superior efficacy in comparison to their respective comparators, as evidenced by reducing the annual relapse rate, MRI inflammatory activity and MRI activity. However, effects on disability progression were heterogeneous across trials and not consistently significant. In patients with more active RRMS, clinical and radiological efficacy was more pronounced, thus supporting the indication of these drugs in this subgroup. It is noteworthy that anti-CD20 drugs (ocrelizumab, ofatumumab and ublituximab) have been shown to minimize acute inflammatory activity, as evidenced by the almost complete reduction of gadolinium-enhancing lesions on MRI, as well as a lower incidence of new or increased lesions on T2. Alemtuzumab and ofatumumab have been shown to have a potential neuroprotective effect suggested by biomarker reductions, as evidenced by a decrease in serum neurofilament concentrations. These concentrations are biomarkers of axonal damage.

Monoclonal antibodies have demonstrated an acceptable safety profile, with the exception of daclizumab. The most frequent adverse events reported were infections and infusion-related reactions.

Despite the evidence available to date, further studies are needed to evaluate the clinical efficacy and safety of these drugs in relation to each other and to other treatments for RRMS. This could be useful when choosing the specific treatment to be individualized on a case-by-case basis.

## Supplementary Information

Below is the link to the electronic supplementary material.

Supplementary file1 (DOCX 140 KB)

## Data Availability

This is a systematic review article, so we do not have original data to share. The main findings of this study were displayed in the figures, tables, and the supplementary material.
