# Supplementary material for: Efficacy and safety of monoclonal antibodies in the treatment of relapsing remitting multiple sclerosis: a systematic review
Source: J Neurol. 2026 Apr 22;273(5):286. doi: 10.1007/s00415-026-13824-y (PMC13102939; doi:10.1007/s00415-026-13824-y)
Supplement: Supplementary file 1 — Supplementary file1 (DOCX 140 KB) [file 415_2026_13824_MOESM1_ESM.docx]

### **Table 1.** **Search Strategy**

| **Database** | **ID** | **Search strategy** |
| --- | --- | --- |
| *Pubmed* |  |  |
|  | #1 | (("multiple sclerosis"[MeSH Terms] OR ("multiple"[All Fields] AND "sclerosis"[All Fields]) OR "multiple sclerosis"[All Fields]) AND (("recurrence"[MeSH Terms] OR "recurrence"[All Fields] OR "relapse"[All Fields] OR "relapses"[All Fields] OR "relapsing"[All Fields] OR "relapsed"[All Fields] OR "relapser"[All Fields] OR "relapsers"[All Fields]) AND ("remit"[All Fields] OR "remits"[All Fields] OR "remitted"[All Fields] OR "remitting"[All Fields])) AND ("antibodies monoclonal"[Supplementary Concept] OR "antibodies monoclonal"[All Fields] OR "monoclonal antibodies"[All Fields] OR "antibodies, monoclonal"[MeSH Terms] OR ("antibodies"[All Fields] AND "monoclonal"[All Fields]) OR ("monoclonal"[All Fields] AND "antibodies"[All Fields]))) AND ((clinicaltrial[Filter] OR randomizedcontrolledtrial[Filter]) AND (2012:2025[pdat])) |
|  | #2 | (("multiple sclerosis"[MeSH Terms] OR ("multiple"[All Fields] AND "sclerosis"[All Fields]) OR "multiple sclerosis"[All Fields]) AND (("recurrence"[MeSH Terms] OR "recurrence"[All Fields] OR "relapse"[All Fields] OR "relapses"[All Fields] OR "relapsing"[All Fields] OR "relapsed"[All Fields] OR "relapser"[All Fields] OR "relapsers"[All Fields]) AND ("remit"[All Fields] OR "remits"[All Fields] OR "remitted"[All Fields] OR "remitting"[All Fields])) AND ("ocrelizumab"[Supplementary Concept] OR "ocrelizumab"[All Fields])) AND ((clinicaltrial[Filter] OR randomizedcontrolledtrial[Filter]) AND (2012:2025[pdat])) |
|  | #3 | (("multiple sclerosis"[MeSH Terms] OR ("multiple"[All Fields] AND "sclerosis"[All Fields]) OR "multiple sclerosis"[All Fields]) AND (("recurrence"[MeSH Terms] OR "recurrence"[All Fields] OR "relapse"[All Fields] OR "relapses"[All Fields] OR "relapsing"[All Fields] OR "relapsed"[All Fields] OR "relapser"[All Fields] OR "relapsers"[All Fields]) AND ("remit"[All Fields] OR "remits"[All Fields] OR "remitted"[All Fields] OR "remitting"[All Fields])) AND ("ofatumumab"[Supplementary Concept] OR "ofatumumab"[All Fields])) AND ((clinicaltrial[Filter] OR randomizedcontrolledtrial[Filter]) AND (2012:2025[pdat])) |
|  | #4 | (("multiple sclerosis"[MeSH Terms] OR ("multiple"[All Fields] AND "sclerosis"[All Fields]) OR "multiple sclerosis"[All Fields]) AND (("recurrence"[MeSH Terms] OR "recurrence"[All Fields] OR "relapse"[All Fields] OR "relapses"[All Fields] OR "relapsing"[All Fields] OR "relapsed"[All Fields] OR "relapser"[All Fields] OR "relapsers"[All Fields]) AND ("remit"[All Fields] OR "remits"[All Fields] OR "remitted"[All Fields] OR "remitting"[All Fields])) AND ("ublituximab"[Supplementary Concept] OR "ublituximab"[All Fields])) AND ((clinicaltrial[Filter] OR randomizedcontrolledtrial[Filter]) AND (2012:2025[pdat])) |
|  | #5 | (("multiple sclerosis"[MeSH Terms] OR ("multiple"[All Fields] AND "sclerosis"[All Fields]) OR "multiple sclerosis"[All Fields]) AND (("recurrence"[MeSH Terms] OR "recurrence"[All Fields] OR "relapse"[All Fields] OR "relapses"[All Fields] OR "relapsing"[All Fields] OR "relapsed"[All Fields] OR "relapser"[All Fields] OR "relapsers"[All Fields]) AND ("remit"[All Fields] OR "remits"[All Fields] OR "remitted"[All Fields] OR "remitting"[All Fields])) AND ("rituximab"[Supplementary Concept] OR "rituximab"[All Fields] OR "rituximab"[MeSH Terms] OR "rituximab s"[All Fields])) AND ((clinicaltrial[Filter] OR randomizedcontrolledtrial[Filter]) AND (2012:2025[pdat])) |
|  | #6 | (("multiple sclerosis"[MeSH Terms] OR ("multiple"[All Fields] AND "sclerosis"[All Fields]) OR "multiple sclerosis"[All Fields]) AND (("recurrence"[MeSH Terms] OR "recurrence"[All Fields] OR "relapse"[All Fields] OR "relapses"[All Fields] OR "relapsing"[All Fields] OR "relapsed"[All Fields] OR "relapser"[All Fields] OR "relapsers"[All Fields]) AND ("remit"[All Fields] OR "remits"[All Fields] OR "remitted"[All Fields] OR "remitting"[All Fields])) AND ("natalizumab"[Supplementary Concept] OR "natalizumab"[All Fields] OR "natalizumab"[MeSH Terms] OR "natalizumab s"[All Fields])) AND ((clinicaltrial[Filter] OR randomizedcontrolledtrial[Filter]) AND (2012:2025[pdat])) |
|  | #7 | (("multiple sclerosis"[MeSH Terms] OR ("multiple"[All Fields] AND "sclerosis"[All Fields]) OR "multiple sclerosis"[All Fields]) AND (("recurrence"[MeSH Terms] OR "recurrence"[All Fields] OR "relapse"[All Fields] OR "relapses"[All Fields] OR "relapsing"[All Fields] OR "relapsed"[All Fields] OR "relapser"[All Fields] OR "relapsers"[All Fields]) AND ("remit"[All Fields] OR "remits"[All Fields] OR "remitted"[All Fields] OR "remitting"[All Fields])) AND ("alemtuzumab"[Supplementary Concept] OR "alemtuzumab"[All Fields] OR "alemtuzumab"[MeSH Terms] OR "alemtuzumab s"[All Fields])) AND ((clinicaltrial[Filter] OR randomizedcontrolledtrial[Filter]) AND (2012:2025[pdat])) |
|  | #8 | (("multiple sclerosis"[MeSH Terms] OR ("multiple"[All Fields] AND "sclerosis"[All Fields]) OR "multiple sclerosis"[All Fields]) AND (("recurrence"[MeSH Terms] OR "recurrence"[All Fields] OR "relapse"[All Fields] OR "relapses"[All Fields] OR "relapsing"[All Fields] OR "relapsed"[All Fields] OR "relapser"[All Fields] OR "relapsers"[All Fields]) AND ("remit"[All Fields] OR "remits"[All Fields] OR "remitted"[All Fields] OR "remitting"[All Fields])) AND ("daclizumab"[Supplementary Concept] OR "daclizumab"[All Fields] OR "daclizumab"[MeSH Terms])) AND ((clinicaltrial[Filter] OR randomizedcontrolledtrial[Filter]) AND (2012:2025[pdat])) |
| *Cochrane* |  |  |
|  | #1 | (Multiple Sclerosis):ti,ab,kw AND (Relapsing Remitting):ti,ab,kw AND (Monoclonal Antibodies):ti,ab,kw published between 2012 and 2025, with a publication date in the Cochrane Library between January 2012 and December 2025, under ‘Trials’ (variations of the word have been searched for) |
|  | #2 | (Multiple Sclerosis):ti,ab,kw AND (Relapsing Remitting):ti,ab,kw AND (Ocrelizumab):ti,ab,kw published between 2012 and 2025, with a publication date in the Cochrane Library between January 2012 and December 2025, under ‘Trials’ (variations of the word have been searched for) |
|  | #3 | (Multiple Sclerosis):ti,ab,kw AND (Relapsing Remitting):ti,ab,kw AND (Ofatumumab):ti,ab,kw published between 2012 and 2025, with a publication date in the Cochrane Library between January 2012 and December 2025, under ‘Trials’ (variations of the word have been searched for) |
|  | #4 | (Multiple Sclerosis):ti,ab,kw AND (Relapsing Remitting):ti,ab,kw AND (Ublituximab):ti,ab,kw published between 2012 and 2025, with a publication date in the Cochrane Library between January 2012 and December 2025, under ‘Trials’ (variations of the word have been searched for) |
|  | #5 | (Multiple Sclerosis):ti,ab,kw AND (Relapsing Remitting):ti,ab,kw AND (Rituximab):ti,ab,kw published between 2012 and 2025, with a publication date in the Cochrane Library between January 2012 and December 2025, under ‘Trials’ (variations of the word have been searched for) |
|  | #6 | (Multiple Sclerosis):ti,ab,kw AND (Relapsing Remitting):ti,ab,kw AND (Natalizumab):ti,ab,kw published between 2012 and 2025, with a publication date in the Cochrane Library between January 2012 and December 2025, under ‘Trials’ (variations of the word have been searched for) |
|  | #7 | (Multiple Sclerosis):ti,ab,kw AND (Relapsing Remitting):ti,ab,kw AND (Alemtuzumab):ti,ab,kw published between 2012 and 2025, with a publication date in the Cochrane Library between January 2012 and December 2025, under ‘Trials’ (variations of the word have been searched for) |
|  | #8 | (Multiple Sclerosis):ti,ab,kw AND (Relapsing Remitting):ti,ab,kw AND (Daclizumab):ti,ab,kw published between 2012 and 2025, with a publication date in the Cochrane Library between January 2012 and December 2025, under ‘Trials’ (variations of the word have been searched for) |
| *WOS* |  |  |
|  | #1 | (TS=(Multiple Sclerosis) AND ALL=(Relapsing Remitting) AND ALL=(Monoclonal Antibodies)) AND (DT==("ARTICLE")) Time period: 2012-01-01 to 2025-12-31 |
|  | #2 | (TS=(Multiple Sclerosis) AND ALL=(Relapsing Remitting) AND ALL=(Ocrelizumab)) AND (DT==("ARTICLE")) Time period: 2012-01-01 to 2025-12-31 |
|  | #3 | (TS=(Multiple Sclerosis) AND ALL=(Relapsing Remitting) AND ALL=(Ofatumumab)) AND (DT==("ARTICLE")) Time period: 2012-01-01 to 2025-12-31 |
|  | #4 | (TS=(Multiple Sclerosis) AND ALL=(Relapsing Remitting) AND ALL=(Ublituximab)) AND (DT==("ARTICLE")) Time period: 2012-01-01 to 2025-12-31 |
|  | #5 | (TS=(Multiple Sclerosis) AND ALL=(Relapsing Remitting) AND ALL=(Rituximab)) AND (DT==("ARTICLE")) Time period: 2012-01-01 to 2025-12-31 |
|  | #6 | (TS=(Multiple Sclerosis) AND ALL=(Relapsing Remitting) AND ALL=(Natalizumab)) AND (DT==("ARTICLE")) Time period: 2012-01-01 to 2025-12-31 |
|  | #7 | (TS=(Multiple Sclerosis) AND ALL=(Relapsing Remitting) AND ALL=(Alemtuzumab)) AND (DT==("ARTICLE")) Time period: 2012-01-01 to 2025-12-31 |
|  | #8 | (TS=(Multiple Sclerosis) AND ALL=(Relapsing Remitting) AND ALL=(Daclizumab)) AND (DT==("ARTICLE")) Time period: 2012-01-01 to 2025-12-31 |

**Table 2**. GRADE for domains associated with the efficacy and safety of monoclonal antibody treatment in RRMS.

| **Evidence domain** | **No. of studies** | **Risk of bias** | **Inconsistency** | **Indirectness** | **Imprecision** | **Publication bias** | **Quality of GRADE** |
| --- | --- | --- | --- | --- | --- | --- | --- |
| Adverse events | 1 [24] | Serious limitation | Not serious | Not serious | Not serious | Not serious | Moderate |
| Confirmed disability accumulation | 9 [19,21-23, 25-29] | Serious limitation | Not serious | Not serious | Not serious | Not serious | Moderate |
| Confirmed disability improvement | 4 [21,25,27,29] | Serious limitation | Not serious | Not serious | Not serious | Not serious | Moderate |
| Relapse rate | 10 [19,20,22-29] | Serious limitation | Not serious | Not serious | Not serious | Not serious | Moderate |
| Serum neurofilament light chain | 3 [20,27,28] | Serious limitation | Not serious | Not serious | Not serious | Not serious | Moderate |
| Symbol Digit Modalities Test | 3 [22,23,29] | Not serious | Not serious | Not serious | Not serious | Not serious | High |

**Figure 1.** Risk of bias of the articles included in the systematic review.


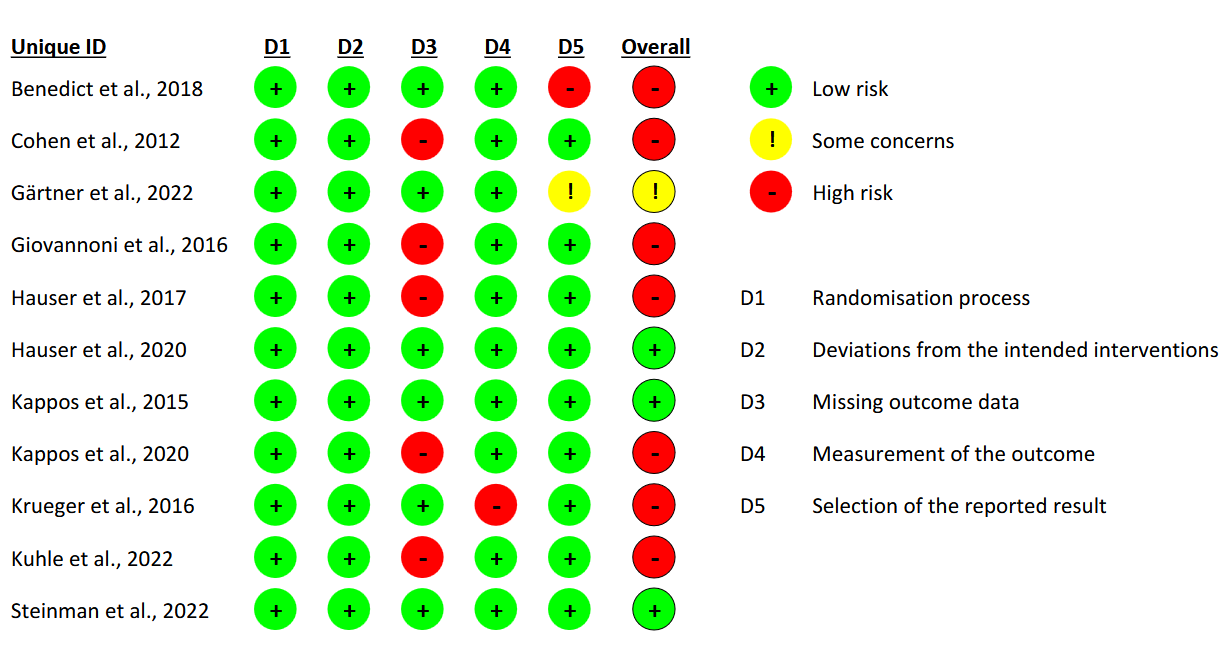


**Table 3**. Detailed description of the percentage of adverse events associated with the monoclonal antibodies included in the study.

| **Adverse Event** | **Alemtuzumab** | **Daclizumab** | | **Ocrelizumab** | | **Ofatumumab** | | | **Ublituximab** | |
| --- | --- | --- | --- | --- | --- | --- | --- | --- | --- | --- |
|  | CARE-MS I [19] | DECIDE [22] | DECIDE [24] | OPERA I [25] | OPERA II [25] | ASCLEPIOS I [27,28] | ASCLEPIOS II  [27,28] | | ULTIMATE I [29] | ULTIMATE II [29] |
| **Any AE** | 96 | 91 |  | 80.1 | 86.3 | 82.2 | 85 | | 86.1 | 92.3 |
| **Serious AE** | 18 | 24 |  | 6.9 | 7 | 10.3 | 7.9 | | 11.4 | 10.3 |
| **Infections** | 67 | 65 |  | 56.9 | 60.2 | 49.2 | 53.8 | | 49.5 | 62.1 |
| Serious infections | 2 | 4 |  | 1.2 | 1.4 | 2.6 | 2.5 | | 5.5 | 4.4 |
| Herpes viral infections | 16 |  |  |  |  | 4.9* | |  |  |  |
| Herpes zoster |  |  |  | 2.2 | 1.9 |  | | |  |  |
| Oral herpes |  |  |  | 2.2 | 3.6 | 2.6* | |  | 0 | 5.1 |
| Nasopharyngitis | 20 | 25 |  |  |  | 18* | |  | 12.5 | 24.3 |
| Urinary tract infections | 17 | 10 |  | 12.7 | 10.6 | 10.3* | |  | 4.0 | 4.0 |
| Upper respiratory tract infection | 15 | 16 |  | 14.5 | 15.8 | 12.7* | |  | 6.2 | 8.8 |
| **Autoimmune events** |  |  |  |  |  |  |  | |  |  |
| **Thyroid disorders** | 18 |  |  |  |  | 1* | |  |  |  |
| **Thyroid cancer** | 1 |  |  |  |  |  |  | |  |  |
| **Blood and lymphatic system disorders** | 18 | 2 |  |  |  | 3.4* | |  | 15.4 | 14.3 |
| **Hepatic events** |  | 16 |  |  |  |  |  | |  |  |
| Liver toxicity | 4 |  |  |  |  |  |  | |  |  |
| **Hepatobiliary disorder** |  | 3 |  |  |  | 1.8* | |  |  |  |
| **Cutaneous events** | 37 | 37 | 37 |  |  |  |  | |  |  |
| Any serious cutaneous AE | 2 | 2 | 2 |  |  |  |  | |  |  |
| Any moderate cutaneous AE |  |  | 14 |  |  |  |  | |  |  |
| Any mild cutaneous AE |  |  | 21 |  |  |  |  | |  |  |
| Rash | 12 |  | 7 |  |  | 2.1* | |  | 0 | 1.1 |
| Dermatitis and eczema |  |  | 14 |  |  | 0.9* | |  |  |  |
| Psoriasis |  |  | 2 |  |  | 0.2* | |  |  |  |
| Alopecia |  |  | 2 |  |  | 5.7* | |  | 2.2 | 4.8 |
| **Infusion-injection reactions** | 90 |  |  | 30.9 | 37.6 | 16.1 | 24.1 | | 44 | 51.5 |
| **Neoplasm** |  |  |  | 0.7 | 0.2 | 0.6**^₷^** | 0.4**^₷^** | | 0 | 0.7 |
| **Number of Deaths** | 1 | 1**^∫^** |  | 0 | 1**^∫^** | 0 | 0 | | 2**^∫^** | 1**^∫^** |

AE: adverse event; ^₷^: neoplasms reported in ASCLEPIOS I were one case of malignant melanoma in situ, one case of invasive breast carcinoma and one case of recurrent non-Hodkings lymphoma. Neoplasms reported in ASCLEPIOS II were two cases of basal-cell carcinoma. None of the malignant events were considered by the investigator to be relates to trial treatment; **∫:** deaths occurring during the DECIDE trial were due to secondary complications of aspiration pneumonia (the investigators not considered to be related to treatment), during the OPERA II trial were due to suicide and during the ULTIMATE I and ULTIMATE II trials were due to pneumonia (deemed to be possibly related to treatment), encephalitis (after measles), and salpingitis (after ectopic pregnancy). *: AEs in participants from the ASCLEPIOS I and II trials (safety analysis set).
